# Effect of Vaginal Laser and Topical Therapies on Vulvovaginal Atrophy Symptoms in Breast Cancer Patients: A Systematic Review and Meta-Analysis

**DOI:** 10.3390/jcm13206131

**Published:** 2024-10-15

**Authors:** Lotti Lúcia Lőczi, Gábor Vleskó, Máté Éliás, Caner Turan, Panna Kajtár, Réka Tóth, Miklós Sipos, Rita Nagy, Péter Hegyi, Nándor Ács, Szabolcs Várbíró, Márton Keszthelyi

**Affiliations:** 1 Department of Obstetrics and Gynecology, Semmelweis University, 1082 Budapest, Hungary; keszthelyi.lotti.lucia@semmelweis.hu (L.L.L.); vlesko.gabor@semmelweis.hu (G.V.); kretschmer47@gmail.hu (M.É.); sipos.miklos.dr@gmail.com (M.S.); acs.nandor@semmelweis.hu (N.Á.); varbiro.szabolcs@semmelweis.hu (S.V.); 2 Centre for Translational Medicine, Semmelweis University, 1082 Budapest, Hungary; c.caner.turan@gmail.com (C.T.); kajpanni@gmail.com (P.K.); rekatoth83@gmail.com (R.T.); nagyrita003@gmail.com (R.N.); hegyi2009@gmail.com (P.H.); 3 Department of Anesthesiology and Intensive Therapy, Semmelweis University, 1082 Budapest, Hungary; 4 Department of Biostatistics, University of Veterinary Medicine, 1078 Budapest, Hungary; 5 Heim Pál National Pediatric Institue, 1089 Budapest, Hungary; 6 Institute for Translational Medicine, Medical School, University of Pécs, 7624 Pécs, Hungary; 7 Institute of Pancreatic Diseases, Semmelweis University, 1083 Budapest, Hungary; 8 Workgroup of Research Management, Doctoral School, Semmelweis University, 1085 Budapest, Hungary

**Keywords:** vulvovaginal atrophy, breast cancer, laser therapy, genitourinary syndrome, dyspareunia

## Abstract

**Background**: Vulvovaginal atrophy (VVA) significantly impacts the quality of life in breast cancer patients leading to symptoms like vaginal dryness, dyspareunia, and genital discomfort. Quality of life in this context is measured using validated scales like the Vaginal Health Index, Visual Analog Scale (VAS), and the Female Sexual Function Index (FSFI). **Methods**: We performed a systematic review and meta-analysis to identify effective treatment options for VVA, including topical estrogen, systemic hormone therapy, vaginal DHEA, ospemifene, and non-hormonal methods like intravaginal laser therapy, moisturizers, and lubricants. A systematic search of four databases (MEDLINE, Scopus, CENTRAL, Embase) identified studies on VVA treatment efficacy in breast cancer patients, yielding 13,039 records, with 32 eligible studies and 8 included in the meta-analysis. **Results**: Significant improvements were found with intravaginal laser therapy, showing notable differences in the Vaginal Health Index (MD = 8.24, *p* < 0.01), dyspareunia (MD = −4.82, *p* = 0.05), and dryness (MD = −5.05, *p* = 0.01). However, no significant changes were observed in FSFI and vaginal pH. Notably only intravaginal laser therapy was included in the meta-analysis, as other treatment options lacked comparable data. Both hormonal and non-hormonal treatments improved quality of life, with laser therapy showing the most substantial effects. **Conclusions**: Intravaginal laser therapy is an effective treatment for VVA symptoms in breast cancer survivors, particularly in improving the Vaginal Health Index and reducing dyspareunia. Despite the strengths of the study, variability among studies, lack of RCT-s and data limitations, especially on long-term effects, present challenges.

## 1. Introduction

Breast cancer is the most frequently diagnosed cancer worldwide, with an estimated 2.26 million cases documented in 2020, and it ranks as the leading cause of cancer deaths in females [1]. Hormone receptor-positive (HR+) breast cancer accounts for roughly 70% of all breast cancer cases. A widely accepted approach is the use of selective estrogen receptor modulators (SERMs), selective estrogen receptor down-regulators (SERDs), and aromatase inhibitors (AIs) [2]. The consequence of these therapies is a reduction of estrogen circulating in the urogenital receptors, often resulting in vulvovaginal atrophy (VVA), which also affects quality of life [3]. Patients often suffer from vaginal dryness, itching, irritation, dyspareunia, and dysuria, collectively known as the genitourinary syndrome of menopause, which together can lead to pain, discomfort and impairment of sexual function, negatively affecting multiple domains of quality of life [4]. Clinical observations have shown that 60% of breast cancer survivors experience vulvovaginal atrophy in post-menopause and 39.4% in pre-menopause after treatment [5]. An objective vaginal assessment is essential and can be complemented by gold-standard methods such as the Vaginal Health Index (VHI) and the Female Sexual Function Index (FSFI) [6]. In most cases, menopausal genitourinary syndrome can be prevented, reduced, or treated in these patients, but this requires early detection and appropriate treatment. In addition to lifestyle changes and non-hormonal treatments such as vaginal moisturizers, gels, and lubricants, vaginal estrogen therapy, ospemifene, topical androgens, intravaginal dehydroepiandrosterone (DHEA), or intravaginal laser therapy (CO_2_, Erbium) may also be effective [4]. The importance of the topic is demonstrated by the fact that there have been a number of previous studies on the recognition, examination, and treatment of symptoms of vulvovaginal atrophy in women with a history of breast cancer [7,8,9,10,11,12,13,14,15,16,17,18,19,20,21,22,23,24,25,26,27]. The aim of this study is to comprehensively evaluate the effectiveness of intravaginal laser therapy and topical treatments in alleviating vulvovaginal atrophy symptoms in women with breast cancer or breast cancer survivors.

## 2. Materials and Methods

The protocol of the study was registered on PROSPERO (CRD42022379866) [28]. We acknowledge that our final study deviates from the originally registered PROSPERO protocol by incorporating self-control studies and adopting the Vaginal Health Index as the primary outcome. These adjustments are explicitly documented in the manuscript to ensure transparency. We report our systematic review and meta-analysis based on the recommendation of the Preferred Reporting Items for Systematic Reviews and Meta-analyses (PRISMA) 2020 Statement.

We followed the recommendations of the Cochrane Handbook for Systematic Reviews of Interventions Version 6.3. We conducted a systematic literature search in four medical databases: MEDLINE (via PubMed), Embase, CENTRAL, and Scopus, from inception to 17 August 2024 using the following search key: (vulvovaginal atrophy OR vaginal atrophy OR genitourinary syndrome OR urogenital atrophy OR genitalia atrophy OR atrophic vaginitis) AND (breast cancer OR breast tumor OR breast cancer treatment OR breast malignancy OR aromatase inhibitor OR tamoxifen OR selective estrogen receptor modulator OR SERM OR Letrozole OR Anastrozole OR Exemestane OR ovarian suppression OR chemotherapy) for all fields in the given search engines. No language or other restrictions were imposed. Our analysis included studies on breast cancer patients with vulvovaginal atrophy who underwent treatment for any type and stage of breast cancer. The following population–intervention–control–outcome (PICO) framework was used:

P—women with breast cancer treatment;
I—hormonal and non-hormonal treatment;
C—placebo or sham treatment;
O—Primary outcome: Vaginal Health Index (VHI), Female Sexual Function Index (FSFI)

Secondary outcome: Dryness, Dyspareunia, Vaginal pH

### 2.1. Selection Process

After the systematic search of databases, duplication removal and selection were conducted according to the PICO criteria. We used EndNote X9, 20.5 reference manager software (Clarivate Analytics, Philadelphia, PA, USA, 2020). Two independent authors (L.L.L. and M.É.) screened the publications separately for title and abstract as well as full text. After removing 951 duplicates, we screened 12,088 articles; 11,974 articles were excluded during the title and abstract selection, and another 63 articles were excluded during the full-text selection. After the full-text selection, another 19 articles were excluded due to the unusability of the data, so 32 articles were selected for the systematic review and meta-analysis. Inter-reviewer agreement was calculated using Cohen’s Kappa (k = 0.84, and k = 0.86 for the first and second steps of the selection, respectively), and any disagreements were resolved by a third reviewer (M.K.).

### 2.2. Data Items

We collected the following data from the eligible articles: first author, year of publication, study type, study location, number of centers included in the study, study design, demographic data, details of treatments received, and data on outcomes for statistical analysis.

The Vaginal Health Index (VHI) score is a numerical measure used to evaluate vaginal health during clinical examinations. Five evaluated parameters collectively contribute to determining the degree of atrophy in the genitourinary tract, with each parameter assigned a score. The total VHI score ranges from 5 to 25, with lower scores indicative of more pronounced urogenital atrophy.

The Female Sexual Function Index (FSFI) is a validated questionnaire used to assess various aspects of female sexual function. It evaluates domains such as desire, arousal, lubrication, orgasm, satisfaction, and pain during sexual activity. The total score ranges from 2 to 36, with higher scores indicating better sexual function.

Dryness and dyspareunia were measured by Visual Analogue Scale. It is a psychometric tool used to measure subjective characteristics or attitudes, which can range from 0 to 10, with 10 being the most painful and 0 being the least painful.

Vaginal pH was measured by a pH test strip. A normal vaginal pH typically falls within the range of 3.8 to 4.5. Values above this or below this can indicate other conditions. A third reviewer (M.K.) resolved the discrepancies.

To assess the quality of the evidence, we followed the recommendation of the “Grades of Recommendation, Assessment, Development, and Evaluation (GRADE)” workgroup [29].

### 2.3. Study Risk of Bias Assessment

The risk of bias assessment in the outcomes was carried out separately by two reviewers (L.L.L. and M.É.) using the revised tool for assessing the risk of bias (ROBINS-I) [28]. A third reviewer (M.K.) resolved any disagreements that arose. Publication bias was intended to be assessed visually by funnel plots and Egger’s test [30].

### 2.4. Synthesis Methods

We extracted or estimated pre- and post-treatment means and standard deviations (SD) and pooled pre- and post-means as well as the differences between before and after values within a study. All statistical analyses were performed in R (R Core Team 2023, v4.2.3) with the meta (Schwarz er 2022, v6.2.1) package using random-effects models with restricted maximum likelihood estimators and the Hartung–Knapp adjustment for 95% confidence intervals (CI) of each study effect and the pooled effect sizes [31]. When the mean and SD values were not provided, quartiles for estimating the mean and SD Lou and Shi methods were used (as implemented in the used meta R package) [32,33].

In our meta-analysis, we exclusively included observational studies to reduce heterogeneity and ensure more consistent results. The decision to focus solely on observational studies allowed us to maintain a more uniform dataset, as differences in study designs, particularly between observational studies and randomized controlled trials, could introduce significant variability in outcomes. As the studies included reported only before and after means but not differences, SDs of before–after changes were missing. We estimated the variance of the difference as the sum of variances of before and after values. This is a conservative estimate as it is unlikely that there is a negative correlation between the two. Still, a sensitivity analysis was performed with imputed correlations varying between 0.1 and 0.9, leading to similar results for all outcomes except pH. However, this analysis in itself was limited and not entirely reliable due to the small number of included studies, therefore, we believe it has no significant impact on the overall findings detailed in this paper.

Findings of the mean pools and the before-and-after meta-analyses were visualized in forest plots.

## 3. Results

### 3.1. Search and Selection, Characteristics of the Included Studies

**Figure 1:**
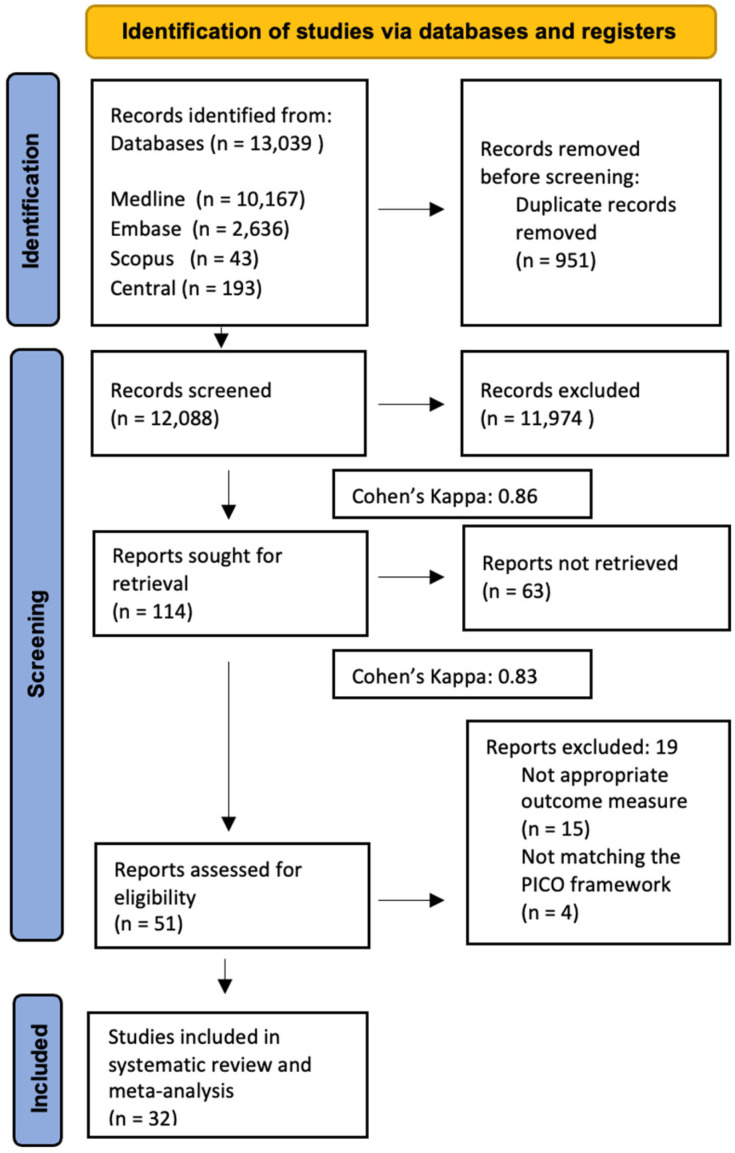
PRISMA Flow Diagram of the screening and selection process.

Our systematic search yielded a total of 13,039 articles; after duplicate removal, title, abstract, and full-text selection, we identified 32 studies (14 randomized trials and 18 observational studies) matching our PICO framework. Despite an extensive systematic search, no controlled studies met the criteria for inclusion in the meta-analysis. Nevertheless, in the absence of these high-quality studies, articles that documented before-and-after changes were pooled for the meta-analysis. Out of the 30 studies deemed eligible for this research, 24 were analyzed solely through qualitative methods. These studies did not meet the criteria for quantitative analysis but provided valuable descriptive insights and thematic understanding. The remaining 8 studies, however, fit the framework for quantitative analysis and were thus included in statistical evaluations to measure and compare numerical data. The results of the other 24 articles are discussed as part of the qualitative synthesis. The summary of the selection process is shown in Figure 1.

### 3.2. Basic Characteristics of Included Studies

The characteristics of the included studies are detailed in Table 1. Studies included in the meta-analysis are the laser treatment group: five studies used the CO_2_-laser (SmartXide2 V2LR, Monalisa Touch, DEKA, Florence, Italy), and three studies used the Er:YAG-laser (Fotona SmoothTM XS, Fotona, Ljubljana Slovenia). The laser treatment papers exhibited sufficient similarity to qualify for a pooled analysis.

All study treatment protocols involved three sessions of intravaginal laser therapies. Outcomes were assessed before the initiation of intravaginal laser therapy and after the last intravaginal laser therapy. The period between the last intravaginal laser therapy and the assessment of the outcomes was defined as the follow-up period. Our study included only studies using the Visual Analogue Scale (VAS) ranging from 0 to 10 to evaluate dyspareunia, dryness, itching, and burning symptoms.

In the sham treatment group, many different preparations were investigated, such as estriol cream, hyaluronic acid gel, testosterone vaginal cream, vaginal moisturizer etc., which are detailed in Table 1. Due to the difference in the intervention groups, these articles are part of the systematic review.

### 3.3. Outcomes

#### 3.3.1. Primary Outcome: Vaginal Health Index

Three studies evaluated post-treatment VHI scores 8 weeks after the first treatment session, one study did so 4 weeks after the first treatment session, one study did so 12 weeks after the first treatment session, and three studies did so at 6 months [8,9,13,17,22,23,24,26].

**Figure 2:**
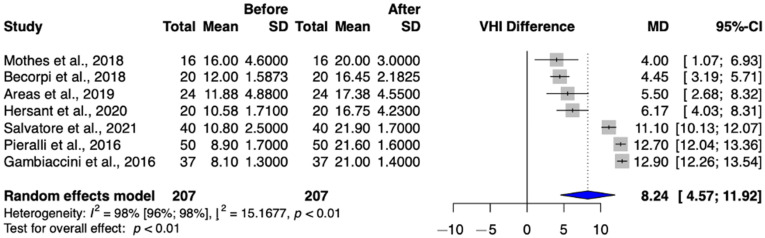
Vaginal Health Index values in the intravaginal laser-treated groups [8,9,13,17,22,24,26].

A total of seven studies (Arêas et al.; Becorpi et al.; Gambacciani et al.; Hersant et al.; Mothes et al.; Pieralli et al.; Salvatore et al.) were selected for analysis, involving a total of 207 patients [8,9,13,17,22,24,26]. VHI significantly increased after treatment (MD = 8.24 (95% CI: 4.57–11.92) *p*-value < 0.01) (Figure 2).

#### 3.3.2. Primary Outcome: Female Sexual Function Index (FSFI)

A total of three studies (Becorpi et al.; Pearson et al.; Salvatore et al.) were selected for analysis, involving a total of 71 patients [9,23,26]. However, due to inconsistencies in scoring methodologies, the inclusion of an out-of-range FSFI score, and significant heterogeneity between study populations, the data were only partially compatible (Supplementary Figure S2).

A further 24 articles were found eligible for qualitative synthesis [7,10,11,12,14,15,16,18,19,20,21,27,34,35,36,37,38,39,40,41,42,43,44]. Due to inconsistent interventions applied in the study designs, we opted to exclude them from the meta-analysis to be able to generate a higher level of evidence. Overall, these studies show significant improvements in VHI and in FSFI Scores after treatment. Results from non-laser therapies showed that patient complaints were reduced, but this effect was achieved over a longer period of time than with laser therapy. However, these results are not easily generalizable to current clinical practice.

### 3.4. Secondary Outcome

#### 3.4.1. Secondary Outcome: Dyspareunia

**Figure 3:**
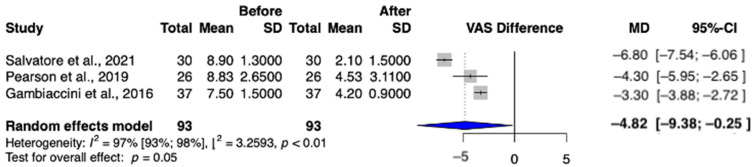
Dyspareunia values in the intravaginal laser-treated groups [13,23,26].

A total of three studies (Gambacciani et al.; Pearson et al.; Salvatore et al.) were selected for analysis, involving a total of 93 patients [13,23,26]. In the post-treatment group, the incidence of dyspareunia was statistically significant (MD = −4.82 (95% CI: −9.38–−0.25) *p* = 0.05) (Figure 3).

#### 3.4.2. Secondary Outcome: Dryness

**Figure 4:**
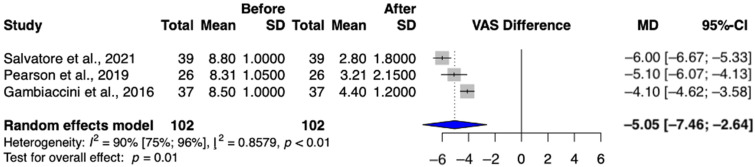
Dryness values in the intravaginal laser-treated groups [13,23,26].

A total of three studies (Gambacciani et al.; Pearson et al.; Salvatore et al.) were selected for analysis, involving a total of 102 patients [13,23,26]. The dryness score assessed by using the Visual Analog Scale was significantly lower after intravaginal laser treatment (MD = −5.05 (95% CI: −7.46–−2.64) *p* = 0.01) (Figure 4).

#### 3.4.3. Secondary Outcome: Vaginal pH

**Figure 5:**
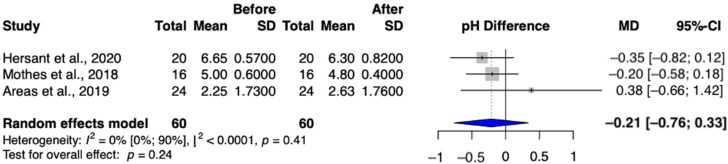
Vaginal pH values in the intravaginal laser-treated groups [8,17,22].

A total of three studies (Arêas et al.; Hersant et al.; Mothes et al.) were selected to analyze changes in vaginal pH, involving a total of 60 patients [8,17,22]. After treatment, the vaginal pH did not significantly decrease (MD = −0.21 (95% CI: −0.76–−0.33) (Figure 5).

### 3.5. Risk of Bias and Level of Evidence Certainty Assessments

The risk of bias assessment for the studies included in the meta-analysis and systematic review is presented in Figure S1 in the Supplementary Material. None of the studies in the meta-analysis exhibited a high risk of bias. However, some concerns about bias were raised due to confounding across all articles. The risks of bias associated with deviation from the intended intervention, missing outcome data, and selection of reported results domains were all found to be low. The only outcome suitable for a funnel plot is the Vaginal Health Index with seven studies. However, the limited number of studies prevents a reliable Egger’s test. While the VHI funnel plot was examined, its scattered distribution and asymmetry, with studies at the extremes, make interpretation difficult. This likely reflects the heterogeneity of the included studies rather than clear evidence of publication bias. The funnel plot is presented in Figure S2 in the Supplementary Material.

In most outcomes, the GRADE assessment results were “low quality”, as only observational studies were included in the analysis.

## 4. Discussion

This systematic review and meta-analysis evaluated different treatment methods for vulvovaginal atrophy caused by anti-estrogen therapy in breast cancer patients. Our aim was to provide an evidence-based estimate of the effectiveness of treatment for vulvovaginal atrophy among breast cancer patients.

The use of topical treatments and intravaginal laser therapy is recommended for the management of moderate to severe VVA in women with breast cancer [4].

The importance of vulvovaginal atrophy therapy plays a crucial role in improving the quality of life of cancer patients. Among the symptoms of VVA, vaginal dryness is frequently experienced by 21–47% of postmenopausal women and plays a role in sexual dysfunction, causing significant socio-sanitary issues [45,46].

Research data suggests that individuals who are long-term survivors of breast cancer generally experience normalization in terms of physical and emotional functioning. Nonetheless, they continue to experience difficulties with sexual functioning and satisfaction for at least 5 years following treatment [45].

FSFI and VHI have been extensively researched and validated as important indicators of sexual quality of life [47]. Enhancing these scores has been shown to improve women’s sexual well-being. However, it is crucial to acknowledge that sexual quality of life depends on more than just these markers. Previous studies have demonstrated that laser treatments do not affect libido, likely due to its complexity and the stronger influence of psychological and relational factors [6,8,48].

Cultural background significantly affects how women perceive and report sexual health issues, such as symptoms of vulvovaginal atrophy [49]. Different cultural norms around discussing sexual health may influence the reporting of symptom severity and treatment outcomes [50,51]. This variability could have impacted our findings, as cultural factors may lead to underreporting or different interpretations of symptoms. Future studies should consider these influences to enhance the generalizability of results by using culturally adaptive tools to capture more accurate perceptions of sexual function across diverse populations.

Our results support the outcomes of earlier studies and suggest that the implementation of intravaginal laser and topical therapies can alleviate symptoms of vulvovaginal atrophy. Consequently, this intervention can offer a better quality of life for patients whose daily lives are affected by this condition.

It is important to note that the majority of eligible studies have experienced a drawback due to the limited follow-up duration. It is important to highlight that while several studies demonstrated positive outcomes for intravaginal laser treatments, nearly half of the randomized controlled trials included in our analysis did not show significant differences in improvement compared to control groups [13,14,22,25,26,27].

In the management of vulvovaginal atrophy in women at risk of breast cancer, CO_2_ or Er:Yag lasers offer hormone-free alternatives, with potential advantages over pharmacological therapies.

Studies using intravaginal laser therapy have reached the same conclusion: it is effective in improving symptoms of vulvovaginal atrophy [8,9,13,15,17,22,23,25,26]. These results have been confirmed by two studies examining topical creams containing estrogen [10,18].

Regarding the Vaginal Health Index, we found that our results reassured the conclusion of previous studies, as we found that patient values improved significantly after intravaginal laser treatment, crossing the threshold of atrophy and approaching physiological values [8,9,13,17,22,24,26]. Similar outcomes were confirmed in a study involving Platelet Rich Plasma and hyaluronic acid injection content [16].

This consistently positive finding observed in laser studies may prompt clinicians to consider recommending this method.

We observed no significant change in FSFI after intravaginal laser treatment. However, patient values did increase, indicating a potential positive shift in the treatment satisfaction [9,23,26].

Several topical creams have also achieved similar results, including local testosterone preparations and hyaluronic acid therapy, although not all studies addressed the improvement of this value [34,36].

Dyspareunia, which is defined as pain during sexual intercourse, was measured by VAS, and our results show a significant reduction in pain as a result of the treatment, indicating that the quality of the sexual life of patients improved [13,23,26]. The consistently positive results of intravaginal laser studies might encourage clinicians to consider validating intravaginal laser technology to optimize the sensation of dyspareunia.

Furthermore, there was a reduction in the sensation of dryness among patients, with a mathematically significant reduction in the group receiving intravaginal laser treatment [14,23,26].

Similar effects have been confirmed in numerous articles where the therapy included hyaluronic acid-based vaginal moisturizer, IVT cream, topical lidocaine, olive oil, vaginal exercise, and moisturizer as the examined substances [7,11,38,40].

As a result of the intravaginal laser treatment, we found no significant changes in pH values, but their clinical significance is relevant because different pH values of patients started to approach the normal range, which also indicated an improvement in vaginal flora.

Physical therapies offer a promising complement to laser and topical treatments for managing VVA. Interventions such as vaginal dilators and pelvic floor therapy, which address muscle dysfunction and dyspareunia, show potential benefits [52]. While less studied in breast cancer patients, evidence from postmenopausal women suggests these therapies improve symptoms like dyspareunia and incontinence. Given these positive outcomes, similar approaches may benefit breast cancer patients, but further research is needed to confirm their effectiveness in this special group [45,52,53,54].

As outlined in the American Cancer Society/American Society of Clinical Oncology Breast Cancer Survivorship Care Guideline, primary care clinicians are encouraged to thoroughly assess patients for potential sexual dysfunction or difficulties with sexual intimacy. Once identified, they should detect and address any reversible factors contributing to these issues. Moreover, clinicians should offer non-hormonal, water-based lubricants and moisturizers to alleviate vaginal dryness. When necessary, patients should be referred to specialized services such as psychoeducational support, group therapy, sexual counseling, marital counseling, or intensive psychotherapy to help them address their specific concerns appropriately [55].

Vaginal laser treatment offers a promising, cutting-edge, and low-impact approach to addressing symptoms of vulvovaginal atrophy. Extensive research supports its efficacy, making it a viable therapeutic option. Given the current limitations in high-quality evidence, laser treatment for vulvovaginal atrophy should remain primarily at a research level until further rigorous studies can confirm its long-term effectiveness and safety for broader clinical use.

A number of studies in the literature have consistently observed remarkable improvements in vulvovaginal symptoms and sexual function. These positive effects are evident both immediately after treatment and during long-term follow-ups. Although topical treatments indicate improvement in certain values, laser therapy shows improvement in all monitored parameters [8,9,15,17,23,24].

On the basis of the available data, it appears that laser intervention to induce morphological changes in vaginal tissue can alleviate symptoms associated with vaginal dryness and dyspareunia in the genitourinary syndrome of menopause. According to the consensus recommendations of the North American Menopause Society and the International Society for the Study of Women’s Sexual Health, when addressing GSM in women with or at high risk of breast cancer, microablative fractional CO_2_ laser or non-ablative vaginal Erbium YAG laser (VEL) are viable alternatives that eliminate the need for hormone-based treatments, which can be more beneficial compared to pharmacologic therapies. Although the CO_2_ laser is believed to primarily target superficial tissue, VEL appears to remodel deeper collagen and stimulate collagen synthesis. This effect has the potential to stimulate the production of new collagen, leading to enhanced tissue integrity and elasticity over time [9].

### 4.1. Strengths and Limitations

The strengths of this article lie in the comprehensive literature review, which covers a wide range of studies on vulvovaginal atrophy in breast cancer patients. It adheres to transparent reporting guidelines, utilizes robust risk of bias and quality of evidence assessments, and provides practical recommendations for clinical practice. Together, these elements enhance the credibility and clinical relevance of study findings. Our primary limitation is the substantial variability among the included studies. These studies varied in quality, encompassing observational studies, self-controlled before-and-after studies, and randomized controlled trials. The amalgamation of these heterogeneous study designs may have introduced variability, potentially diminishing the overall reliability and robustness of the findings. The other limitation is that the number of studies included in our analysis was insufficient to yield reliable or meaningful results from subgroup or sensitivity analyses. In addition, there is a limitation of data, particularly regarding long-term treatment effects. These studies should track patients for several years to assess whether the therapeutic effects persist and to identify any long-term adverse effects. This approach will provide a clearer picture of the sustainability of treatment benefits over time.

Another limitation of our study is the lack of detailed data on potential confounders like cancer treatments, hormonal status, and treatment adherence. These unreported variables may influence the outcomes of vaginal laser therapy and topical treatments. Since we were unable to account for these confounders, future studies should prioritize collecting this information to improve the validity and generalizability of findings. We acknowledge that many of the included studies were industry-sponsored, which may have influenced the reported outcomes, highlighting the need for independent research to minimize potential conflicts of interest.

### 4.2. Implications for Practice

For both patients and clinicians, it is very important that intervention against VVA can reduce the decline in sexual function, thereby improving quality of life. The unquestionable importance of the immediate application of research findings in clinical practice is widely acknowledged (AE articles). In line with the philosophy of translational medicine, our study was diligently designed to bridge the gap between scientific research and practical bedside application [56,57].

Our aim was to provide valuable insights and recommendations that can directly benefit patients.

### 4.3. Implications for Research

In the pursuit of advancing our understanding of vulvovaginal atrophy management in breast cancer patients, future research endeavors should consider several critical areas for improvement. There remains a notable need for more RCTs and long-term studies in this field. We recommend conducting randomized controlled trials with larger sample sizes, extended follow-up periods, and a more diverse patient population. Such studies would provide more robust evidence and improve the generalizability of the findings. Additionally, incorporating a variety of demographics and clinical presentations could help capture a broader spectrum of treatment responses and adverse effects. Exploring the integration of current therapies with complementary interventions, such as physical therapy or psychological support, could also enhance the management of vulvovaginal atrophy. This multidisciplinary approach might improve overall treatment efficacy and patient satisfaction by addressing both the physical and psychosocial aspects of the condition. Furthermore, future research should investigate the long-term effects of combined treatment modalities to understand their safety and effectiveness over time. Such comprehensive investigations are crucial for developing more effective and personalized therapeutic strategies for VVA. These studies would enhance the overall quality of research, but they would also provide a stronger basis for clinical decision-making and highlight the lasting impact of treatments, ensuring that patients receive the most effective and durable interventions. These efforts will not only contribute to knowledge in this field but will also provide patients and clinicians with a more robust basis for decision-making and treatment strategies. Given the current constraints of available therapies in the effectiveness, safety, and patient adherence, it becomes imperative to explore novel approaches that can prove valuable in this area, and tailoring treatments is of utmost importance.

## 5. Conclusions

Our results highlight that vaginal health shows improvement mostly in the laser group. Topical hormonal and non-hormonal treatments show less progress compared to laser therapy. The optimal duration of therapy for all these treatments remains uncertain.

## Figures and Tables

**Table 1:** Characteristics of studies included.

Author (Year)	Country	Study Type	Included in the Meta-Analysis	Number of Patients	Age (Year, Mean)	Intervention	Control	Main Outcomes
Advani (2017) [7]	USA	randomized controlled trial	No	57	59	vaginal moisturizer	no therapy	FSFI, MSIQ, FSDS-R, menopausal symptom scale
Areas (2019) [8]	Brazil	open, prospective, therapeutic intervention study	Yes	24	53.7	Er:YAG laser	-	VHI
Becorpi (2018) [9]	Italy	clinical prospective study	Yes	20	58.2	CO_2_ laser	-	VHI, FSFI, FSDS-R
Biglia (2010) [10]	Italy	randomized double-blind study	No	26	NR	Estriol cream or Estadiol tablets	Moisturizer	VSS, PFSF, VHI, KI
Carter (2020) [34]	USA	single-arm, prospective longitudinal trial	No	101	55	HLA-based vaginal moisturizing	-	VAS, VuAS, FSFI, MSCL
Chatsiproios (2019) [35]	Germany	prospective, multicenter, observational study	No	117	52	Oil-in-water emulsion	-	Subjective symptoms
Dahir (2014) [36]	USA	single-arm interventional study	No	12	59.67	Testosterone vaginal cream	-	FSFI
Davis (2018) [11]	Australia	Double-blind, randomized, placebo-controlled trial	No	44	56.4	Testosterone vaginal cream	Placebo cream	FSFI, FSDS-R
Dew (2003) [37]	Australia	Cohort study	No	1472	NR	Estriol cream or estradiol tablets	Other subjects	Menopausal symptoms
Donders (2014) [12]	Belgium, Germany	Open-label bicentric phase I pharmacokinetic study	No	16	57	Estriol and Lactobacillus vaginal tablet	-	Hormone level, Vaginal pH, vaginal symptoms
Gambacciani (2015) [14]	Italy	prospective, cohort study	No	45	NR	Er:YAG or Vaginal Erbium laser	-	VHI, Subjective symptoms
Gambacciani (2016) [13]	Italy	prospective, interventional study	Yes	37	50.8	Er:YAG laser	-	VHI
Goetsch (2014) [38]	USA	randomized, double-blind, controlled study	No	49	55.6	Topical lidocaine	normal saline	Subjective symptoms
Gold (2023) [15]	Austria	randomized, controlled trial	No	43	54	Er:YAG laser	hyaluronic acid suppositories	VHI, quality of life, sexual health
Hersant (2018) [16]	France	phase II clinical trial	No	20	60.8	platelet concentrate combined with hyaluronic acid	-	VHI, FSD
Hersant (2020) [17]	France	prospective monocentric study	Yes	20	56.1	CO_2_ laser	-	VHI, FSD
Hickey (2016) [39]	Australia	randomized, double-blind, crossover trial	No	38	53.1	silicone-based gel	water-based gel	sexual discomfort
Hirschberg (2020) [18]	Spain	phase II, randomized, double-blind, placebo-controlled trial	No	69	59	Estriol vaginal gel	Placebo	FSFI
Juliato (2016) [19]	Brazil	randomized clinical trial	No	52	NR	polyacrylic acid	lubricant	FSFI
Juraskova (2013) [40]	Australia	Phase I/II Study	No	25	51	Olive Oil, Vaginal Exercise, and Moisturizer	-	Sexual Activity Questionnaire Satisfaction subscale of the Female Sexual Function Index
Keshavarzi (2019) [20]	Iran	triple-blind, controlled, randomized clinical trial	No	32	NR	D and E vitamin suppositories	Placebo	VMI, vaginal pH
Lee (2011) [21]	South Korea	randomized, double-blind, placebo-controlled study	No	86	NR	pH balanced gel	placebo	subjective symptoms, VHI, VMI
Loprinzi (1997) [41]	USA	double-blind, crossover, randomized clinical trial	No	45	NR	vaginal moisturizer	placebo	VHI, subjective symptoms
Melisko (2016) [42]	USA	randomized clinical trial	No	76	56	Intravaginal testosterone cream	Estradiol vaginal ring	Sexual Satisfaction
Mension (2023) [27]	Spain	randomized clinical trial	No	72	52.6	CO_2_ laser therapy	Sham laser therapy	FSFI, vaginal pH, Vaginal Health Index, quality of life, body image
Mothes (2018) [22]	Germany	retrospective, single-center cohort study	Yes	16	71	Er:YAG	-	VHI
Pearson (2019) [23]	Australia	prospective interventional study	Yes	26	56	CO_2_ laser	-	FSFI, VVA symptoms
Pfeiler (2011) [43]	Austria	prospective study	No	10	65	Vaginal estriol	-	Vaginal symptoms
Pieralli (2016) [24]	Italy	prospective descriptive cohort study	Yes	50	53.3	CO_2_ laser	-	VHI, symptoms
Quick (2021) [25]	USA	multicenter randomized controlled trial (RCT)	No	18	56.3	CO_2_ laser	-	symptoms, FSFI, UD6 score
Salvatore (2021) [26]	Italy, Greece	prospective cohort study	Yes	40	57.6	CO_2_ laser	-	VHI, VAS, FSFI
Witherby (2011) [44]	USA	phase I/II study	No	21	NR	Testosterone cream	-	Symptoms, VMI

## Data Availability

The datasets used in this study can be found in the full-text articles included in the systematic review and meta-analysis.

## Supplementary Materials

The following supporting information can be downloaded at: https://www.mdpi.com/article/10.3390/jcm13206131/s1, Table S1: PRISMA 2020 checklist. Table S2: PRISMA 2020 for abstracts checklist. Table S3: Summary of Findings. Figure S1: Risk of bias assessment of the included studies (ROBINS-I). Figure S2: Funnel plot of risk of bias assessment. Figure S3: Female Sexual Function Index (FSFI) values in laser-treated groups. The alteration in the Female Sexual Function Index (FSFI) observed within the group subjected to laser treatment. Selection protocol. Supplementary References. Reference [58] is cited in the Supplementary Materials.
