# Supplementary material for: Effect of Vaginal Laser and Topical Therapies on Vulvovaginal Atrophy Symptoms in Breast Cancer Patients: A Systematic Review and Meta-Analysis
Source: J Clin Med. 2024 Oct 15;13(20):6131. doi: 10.3390/jcm13206131 (PMC11508551; doi:10.3390/jcm13206131)
Supplement: Supplementary file 1 [file jcm-13-06131-s001.zip › jcm-3190065-supplementary.pdf]

SUPPLEMENTARY MATERIAL

Title

Effect of Vaginal Laser and Topical Therapies on Vulvovaginal Atrophy Symptoms in Breast Cancer Patients: A Systematic Review And Meta-Analysis

Supplementary Table S1: PRISMA 2020 checklist

Supplementary Table S2: PRISMA 2020 for abstracts checklist

Supplementary Table S3: Summary of Findings Table

Supplementary Figure S1: Risk of bias assessment of the included studies (ROBINS-I)

Supplementary Figure S2: Funnel plot of risk of bias assessment

Supplementary Figure S3: Female Sexual Function Index (FSFI) values in laser-treated groups

The alteration in the Female Sexual Function Index (FSFI) observed within the group subjected to laser treatment.

Selection protocol

Supplementary References

Table S1: PRISMA 2020 checklist

Section and Topic	Item #	Checklist item	Location where item is reported
**TITLE**			
Title	1	Identify the report as a systematic review.	Title
**ABSTRACT**			
Abstract	2	See the PRISMA 2020 for Abstracts checklist.	Sup.mat.
**INTRODUCTION**			
Rationale	3	Describe the rationale for the review in the context of existing knowledge.	4
Objectives	4	Provide an explicit statement of the objective(s) or question(s) the review addresses.	4
**METHODS**			
Eligibility criteria	5	Specify the inclusion and exclusion criteria for the review and how studies were grouped for the syntheses.	5
Information sources	6	Specify all databases, registers, websites, organisations, reference lists and other sources searched or consulted to identify studies. Specify the date when each source was last searched or consulted.	5
Search strategy	7	Present the full search strategies for all databases, registers and websites, including any filters and limits used.	5
Selection process	8	Specify the methods used to decide whether a study met the inclusion criteria of the review, including how many reviewers screened each record and each report retrieved, whether they worked independently, and if applicable, details of automation tools used in the process.	6
Data collection process	9	Specify the methods used to collect data from reports, including how many reviewers collected data from each report, whether they worked independently, any processes for obtaining or confirming data from study investigators, and if applicable, details of automation tools used in the process.	6
Data items	10a	List and define all outcomes for which data were sought. Specify whether all results that were compatible with each outcome domain in each study were sought (e.g. for all measures, time points, analyses), and if not, the methods used to decide which results to collect.	6
	10b	List and define all other variables for which data were sought (e.g. participant and intervention characteristics, funding sources). Describe any assumptions made about any missing or unclear information.	-
Study risk of bias assessment	11	Specify the methods used to assess risk of bias in the included studies, including details of the tool(s) used, how many reviewers assessed each study and whether they worked independently, and if applicable, details of automation tools used in the process.	6
Effect measures	12	Specify for each outcome the effect measure(s) (e.g. risk ratio, mean difference) used in the synthesis or presentation of results.	6, 7
Synthesis methods	13a	Describe the processes used to decide which studies were eligible for each synthesis (e.g. tabulating the study intervention characteristics and comparing against the planned groups for each synthesis (item #5)).	6, 7
	13b	Describe any methods required to prepare the data for presentation or synthesis, such as handling of missing summary statistics, or data conversions.	-

Section and Topic	Item #	Checklist item	Location where item is reported
	13c	Describe any methods used to tabulate or visually display results of individual studies and syntheses.	6, 7
	13d	Describe any methods used to synthesize results and provide a rationale for the choice(s). If meta-analysis was performed, describe the model(s), method(s) to identify the presence and extent of statistical heterogeneity, and software package(s) used.	6, 7
	13e	Describe any methods used to explore possible causes of heterogeneity among study results (e.g. subgroup analysis, meta-regression).	-
	13f	Describe any sensitivity analyses conducted to assess robustness of the synthesized results.	6, 7
Reporting bias assessment	14	Describe any methods used to assess risk of bias due to missing results in a synthesis (arising from reporting biases).	6
Certainty assessment	15	Describe any methods used to assess certainty (or confidence) in the body of evidence for an outcome.	6
**RESULTS**			
Study selection	16a	Describe the results of the search and selection process, from the number of records identified in the search to the number of studies included in the review, ideally using a flow diagram.	7
	16b	Cite studies that might appear to meet the inclusion criteria, but which were excluded, and explain why they were excluded.	7
Study characteristics	17	Cite each included study and present its characteristics.	Table 1
Risk of bias in studies	18	Present assessments of risk of bias for each included study.	Sup. mat.
Results of individual studies	19	For all outcomes, present, for each study: (a) summary statistics for each group (where appropriate) and (b) an effect estimate and its precision (e.g. confidence/credible interval), ideally using structured tables or plots.	-
Results of syntheses	20a	For each synthesis, briefly summarise the characteristics and risk of bias among contributing studies.	-
	20b	Present results of all statistical syntheses conducted. If meta-analysis was done, present for each the summary estimate and its precision (e.g. confidence/credible interval) and measures of statistical heterogeneity. If comparing groups, describe the direction of the effect.	9, 10, 11
	20c	Present results of all investigations of possible causes of heterogeneity among study results.	-
	20d	Present results of all sensitivity analyses conducted to assess the robustness of the synthesized results.	-
Reporting biases	21	Present assessments of risk of bias due to missing results (arising from reporting biases) for each synthesis assessed.	11
Certainty of evidence	22	Present assessments of certainty (or confidence) in the body of evidence for each outcome assessed.	11
**DISCUSSION**			
Discussion	23a	Provide a general interpretation of the results in the context of other evidence.	12
	23b	Discuss any limitations of the evidence included in the review.	14
	23c	Discuss any limitations of the review processes used.	14

Section and Topic	Item #	Checklist item	Location where item is reported
	23d	Discuss implications of the results for practice, policy, and future research.	15
**OTHER INFORMATION**			
Registration and protocol	24a	Provide registration information for the review, including register name and registration number, or state that the review was not registered.	5
	24b	Indicate where the review protocol can be accessed, or state that a protocol was not prepared.	-
	24c	Describe and explain any amendments to information provided at registration or in the protocol.	5
Support	25	Describe sources of financial or non-financial support for the review, and the role of the funders or sponsors in the review.	2
Competing interests	26	Declare any competing interests of review authors.	2
Availability of data, code and other materials	27	Report which of the following are publicly available and where they can be found: template data collection forms; data extracted from included studies; data used for all analyses; analytic code; any other materials used in the review.	-

Table S2: PRISMA 2020 for abstracts checklist

Section and Topic	Item #	Checklist item	Reported (Yes/No)
**TITLE**			
Title	1	Identify the report as a systematic review.	Yes
**BACKGROUND**			
Objectives	2	Provide an explicit statement of the main objective(s) or question(s) the review addresses.	No
**METHODS**			
Eligibility criteria	3	Specify the inclusion and exclusion criteria for the review.	No
Information sources	4	Specify the information sources (e.g. databases, registers) used to identify studies and the date when each was last searched.	Yes
Risk of bias	5	Specify the methods used to assess risk of bias in the included studies.	No
Synthesis of results	6	Specify the methods used to present and synthesise results.	No

Section and Topic	Item #	Checklist item	Reported (Yes/No)
**RESULTS**			
Included studies	7	Give the total number of included studies and participants and summarise relevant characteristics of studies.	Yes
Synthesis of results	8	Present results for main outcomes, preferably indicating the number of included studies and participants for each. If meta-analysis was done, report the summary estimate and confidence/credible interval. If comparing groups, indicate the direction of the effect (i.e. which group is favoured).	Yes
**DISCUSSION**			
Limitations of evidence	9	Provide a brief summary of the limitations of the evidence included in the review (e.g. study risk of bias, inconsistency and imprecision).	No
Interpretation	10	Provide a general interpretation of the results and important implications.	Yes
**OTHER**			
Funding	11	Specify the primary source of funding for the review.	No
Registration	12	Provide the register name and registration number.	No

From: Page MJ, McKenzie JE, Bossuyt PM, Boutron I, Hoffmann TC, Mulrow CD, et al. The PRISMA 2020 statement: an updated guideline for reporting systematic reviews. BMJ 2021;372:n71. doi: 10.1136/bmj.n71

Table S3: Summary of Findings Table

Author (year)	Study design	Number of patients	Treatments	Interpretation
Advani (2017)[1]	randomized pilot trial	57	No additional therapy vs hyaluronic acid- based vaginal moisturizer and prebiotic vaginal moisturizer	Active treatment showed notably lower distress regarding sexual function and reported experiencing less dyspareunia compared to those receiving no additional therapy.
Biglia (2010)[2]	preliminary study	26	Estriol cream 0.25 mg or estradiol tablets 12.5 mg	The application of low-dose vaginal estrogen therapy, using either E3 cream or E2 tablets, demonstrates efficacy in alleviating vaginal atrophy among postmenopausal breast cancer survivors without leading to notable fluctuations in serum estrogen levels.
Carter (2020)[3]	single-arm, prospective longitudinal trial	101	HLA-based vaginal moisturizing	Significant enhancements in the symptoms related to the quality of vaginal and vulvar tissues. The FSFI also improved.
Chatsiproios (2019)[4]	prospective, multicenter, observation al study	117	Oil-in-water emulsion	Over 20% of patients indicated the absence of symptoms, while approximately one-third of women witnessed enhancement or complete cessation of urinary incontinence. This improvement was accompanied by a notably substantial reduction in the severity of objective findings related to vaginal dryness.

Dahir (2014)[5]	Pilot study	12	300 µg testosterone vaginal cream	FSFI scores demonstrated notable statistical significance.
Davis (2018)[6]	Double-blind, randomized, placebo-controlled trial	44	IVT cream 300 mg per dose vs identical placebo	Enhanced sexual contentment and diminished dyspareunia.
Dew (2003)[7]	Cohort study	1472	Estriol cream or estradiol tablets	The application of localized vaginal estrogens seems to represent a secure method of hormone treatment in women who have effectively undergone breast cancer treatment and are now encountering bothersome urogenital symptoms.
Donders (2014)[8]	Open label bicentric phase I pharmacokinetic study	16	0.03 mg Estriol and L. acidophilus vaginal tablets	The utilization of Gynoflor vaginal tablets containing 0.03 mg E3 and L. acidophilus appears to be both safe and effective in managing atrophic vaginitis among patients undergoing Aromatase Inhibitor (AI) therapy in BC cases
Gambacciani (2015)[9]	pilot prospective, longitudinal study	45	Er:YAG or Vaginal Erbium laser	After the initial laser treatment, substantial improvements were noted subjectively and objectively. A more prominent effect became evident following the second and third laser sessions.
Goetsch (2014)[10]	randomized, double-blind,	49	Topical lidocain	The targeted application of 4% aqueous lidocaine has proven to be highly beneficial

	controlled study			during in-office assessments of complaints related to genital dryness or pain.
Gold (2023)[11]	randomized clinical trial	43	Er:YAG laser vs hyaluronic acid suppositories	Both treatments—laser therapy and hyaluronic acid therapy—were effective in improving vaginal health, reducing the subjective bother of urogenital atrophy, and enhancing quality of life and sexual health.
Hersant (2018)[12]	phase 2 clinical trial	20	platelet concentrate combined with hyaluronic acid	Injecting PRP combined with HA significantly improves the volume of secretions, elasticity, and epithelial integrity, thereby increasing the overall VHI score.
Hickey (2016)[13]	randomized, double-blind, crossover trial	38	silicone-based gel	A lubricant based on silicone might prove more effective in decreasing overall sexual discomfort during intercourse compared to the water-based alternative.
Hirschberg (2020)[14]	phase II, randomized, double-blind, placebo-controlled trial	69	0.005% estriol vaginal gel	Administering the gel is well-tolerated and leads to enhanced vaginal symptoms and indicators, encompassing vaginal pH and maturation. These improvements are particularly relevant for patients experiencing estrogen depletion, ultimately culminating in enhanced sexual function.
Juliato (2016)[15]	randomized clinical trial	52	polyacrylic acid	Women who received treatment with the polyacrylic acid moisturizer exhibited noteworthy enhancements in sexual desire,

				lubrication, satisfaction, and reported reduced discomfort during sexual intercourse.
Juraskova (2013)[16]	Phase I/II Study	25	Olive Oil, Vaginal Exercise, and Moisturizer	Enhancements in dyspareunia and sexual functionality.
Keshavarzi (2019)[17]	triple-blind, controlled, randomized clinical trial	32	D and E vitamin suppositories	Vaginal suppositories containing vitamin D and E offer advantages by lowering vaginal pH, enhancing the VMI (Vaginal Maturation Index), and ameliorating genitourinary symptoms related to vaginal atrophy in women undergoing tamoxifen treatment for breast cancer.
Lee (2011)[18]	randomized, double-blind, placebo-controlled study	86	vaginal-pH balanced gel	A gel designed to balance vaginal pH has the potential to alleviate vulvovaginal symptoms in breast cancer survivors who have undergone menopause following cancer treatment.
Loprinzi (1997)[19]	double-blind, crossover, randomized clinical trial	45	vaginal moisturizer vs placebo	The placebo employed in the study seems to function as a lubricating substance, whereas Replens seems to adhere to the vaginal mucosa and serve as a more sustained moisturizing agent.
Melisko (2016)[20]	randomized clinical trial	76	Intravaginal testosterone cream vs estradiol vaginal ring	Both an estrogen-releasing vaginal ring and intravaginal testosterone (IVT) demonstrate efficacy in addressing urogenital symptoms among breast cancer patients undergoing AI therapy.

Mension (2023)[21]	randomized clinical trial	72	CO_2 laser therapy vs Sham laser therapy	Vaginal laser treatment was found to be safe. It did not offer greater effectiveness than placebo treatment when used as a first-line therapy in breast cancer survivors receiving aromatase inhibitors.
Pfeiler (2011)[22]	prospective study	10	0.5 mg vaginal estriol	A significant proportion of patients also noted clear enhancements in urogenital issues.
Quick (2021)[23]	pilot, multi-institutional randomized sham-controlled trial	18	CO2 laser	The intervention led to enhanced sexual function in contrast to the sham treatment.
Witherby (2011)[24]	phase I/II pilot study	21	Testosterone cream	A 4-week course of vaginal testosterone demonstrated enhancements in indicators and manifestations of vaginal atrophy linked to AI therapy, all without causing elevated levels of estradiol or testosterone.

Supplementary Figure S1: Risk of bias assessment of the included studies (ROBINS-I)

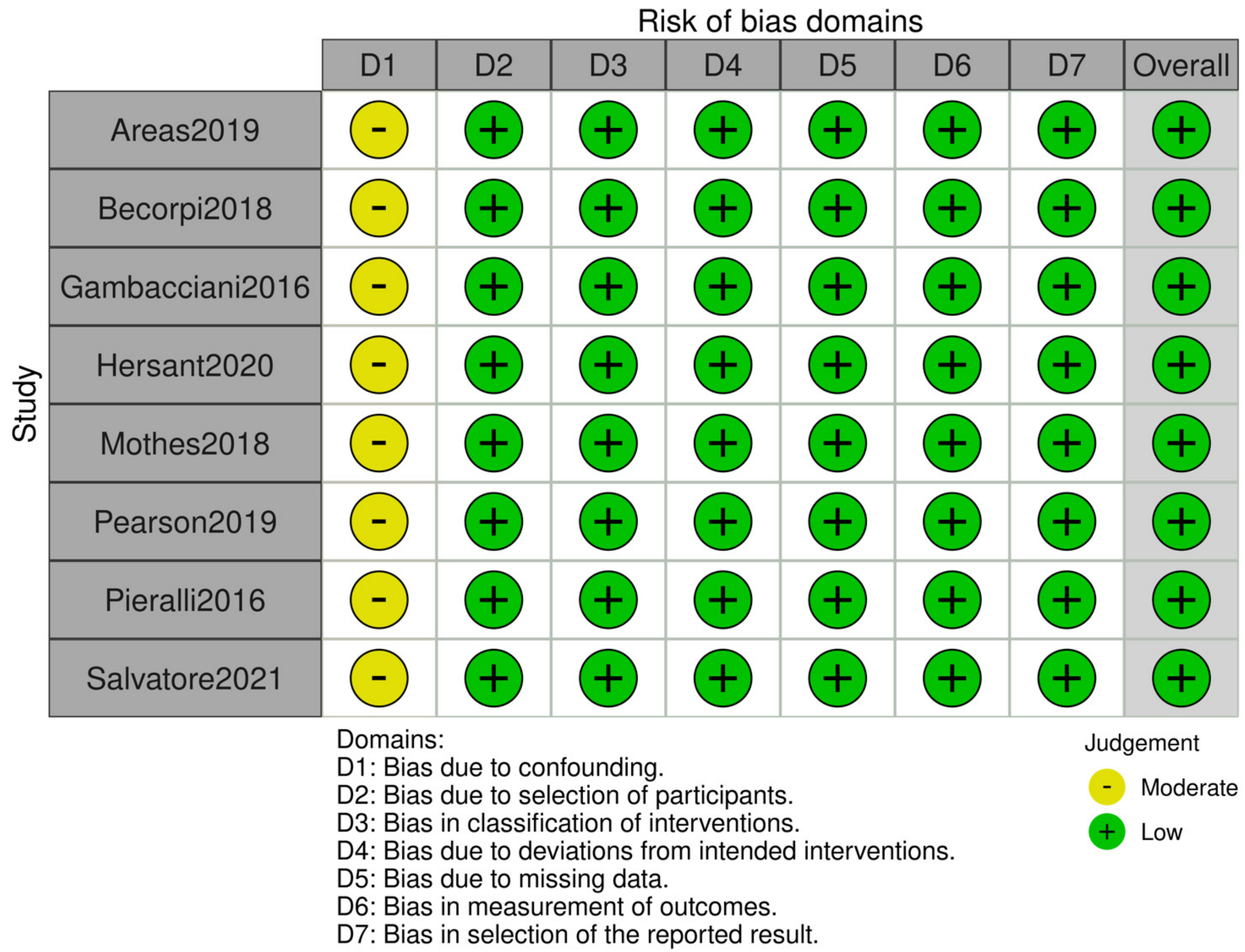

Supplementary Figure S2: Funnel plot of risk of bias assessment

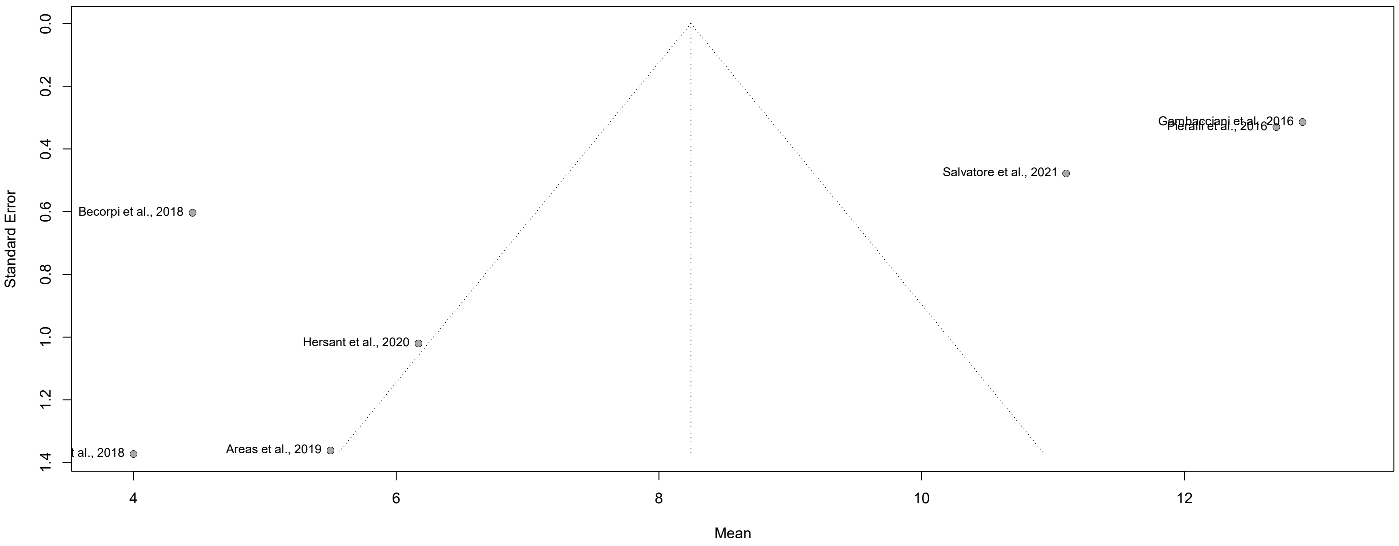

Supplementary Figure S3: Female Sexual Function Index (FSFI) values in laser-treated groups

The alteration in the Female Sexual Function Index (FSFI) observed within the group subjected to laser treatment.

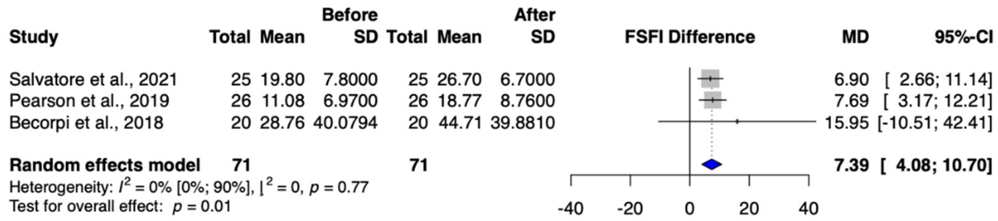

Selection Protocol

Clinical question: What is the most effective treatment for vulvovaginal atrophy in breast cancer treatment?

Title and abstract selection:

Both randomized and non-randomized studies will be included, where adult women with vulvovaginal atrophy underwent treatment for breast cancer of any kind and stage.

Studies or publications with no original research data (such as reviews, letters, commentaries, protocols and so on) will be excluded.

Full text selection:

Groups using the same measurement unit in the tests will be included.

Studies or publications with inappropriate values and not matching the PICO framework will be excluded.

Supplementary References

[1] Advani P, Brewster AM, Baum GP, Schover LR. A pilot randomized trial to prevent sexual dysfunction in postmenopausal breast cancer survivors starting adjuvant aromatase inhibitor therapy. *J Cancer Surviv*. 2017;**11**: 477-85.

[2] Biglia N, Peano E, Sgandurra P, et al. Low-dose vaginal estrogens or vaginal moisturizer in breast cancer survivors with urogenital atrophy: a preliminary study. *Gynecol Endocrinol*. 2010;**26**: 404-12.

[3] Carter J, Baser RE, Goldfrank DJ, et al. A single-arm, prospective trial investigating the effectiveness of a non-hormonal vaginal moisturizer containing hyaluronic acid in postmenopausal cancer survivors. *Support Care Cancer*. 2021;**29**: 311-22.

[4] Chatsiproios D, Schmidts-Winkler IM, König L, Masur C, Abels C. Topical treatment of vaginal dryness with a non-hormonal cream in women undergoing breast cancer treatment - An open prospective multicenter study. *PLoS One*. 2019;**14**: e0210967.

[5] Dahir M, Travers-Gustafson D. Breast cancer, aromatase inhibitor therapy, and sexual functioning: a pilot study of the effects of vaginal testosterone therapy. *Sex Med*. 2014;**2**: 8-15.

[6] Davis SR, Robinson PJ, Jane F, White S, White M, Bell RJ. Intravaginal Testosterone Improves Sexual Satisfaction and Vaginal Symptoms Associated With Aromatase Inhibitors. *J Clin Endocrinol Metab*. 2018;**103**: 4146-54.

[7] Dew JE, Wren BG, Eden JA. A cohort study of topical vaginal estrogen therapy in women previously treated for breast cancer. *Climacteric*. 2003;**6**: 45-52.

[8] Donders G, Neven P, Moegele M, et al. Ultra-low-dose estriol and Lactobacillus acidophilus vaginal tablets (Gynoflor(®)) for vaginal atrophy in postmenopausal breast cancer patients on aromatase inhibitors: pharmacokinetic, safety, and efficacy phase I clinical study. *Breast Cancer Res Treat*. 2014;**145**: 371-9.

[9] Gambacciani M, Levancini M, Cervigni M. Vaginal erbium laser: the second-generation thermotherapy for the genitourinary syndrome of menopause. *Climacteric*. 2015;**18**: 757-63.

[10] Goetsch MF, Lim JY, Caughey AB. Locating pain in breast cancer survivors experiencing dyspareunia: a randomized controlled trial. *Obstet Gynecol*. 2014;**123**: 1231-36.

[11] Gold D, Nicolay L, Avian A, et al. Vaginal laser therapy versus hyaluronic acid suppositories for women with symptoms of urogenital atrophy after treatment for breast cancer: A randomized controlled trial. *Maturitas*. 2023;**167**: 1-7.

[12] Hersant B, SidAhmed-Mezi M, Belkacemi Y, et al. Efficacy of injecting platelet concentrate combined with hyaluronic acid for the treatment of vulvovaginal atrophy in postmenopausal women with history of breast cancer: a phase 2 pilot study. *Menopause*. 2018;**25**: 1124-30.

[13] Hickey M, Marino JL, Braat S, Wong S. A randomized, double-blind, crossover trial comparing a silicone- versus water-based lubricant for sexual discomfort after breast cancer. *Breast Cancer Res Treat*. 2016;**158**: 79-90.

[14] Hirschberg AL, Sánchez-Rovira P, Presa-Lorite J, et al. Efficacy and safety of ultra-low dose 0.005% estriol vaginal gel for the treatment of vulvovaginal atrophy in postmenopausal women with early breast cancer treated with nonsteroidal aromatase inhibitors: a phase II, randomized, double-blind, placebo-controlled trial. *Menopause*. 2020;**27**: 526-34.

[15] Juliato PT, Rodrigues AT, Stahlschmidt R, Juliato CR, Mazzola PG. Can polyacrylic acid treat sexual dysfunction in women with breast cancer receiving tamoxifen? *Climacteric*. 2017;**20**: 62-66.
[16] Juraskova I, Jarvis S, Mok K, et al. The acceptability, feasibility, and efficacy (phase I/II study) of the OVERcome (Olive Oil, Vaginal Exercise, and MoisturizeR) intervention to improve dyspareunia and alleviate sexual problems in women with breast cancer. *J Sex Med*. 2013;**10**: 2549-58.
[17] Keshavarzi Z, Janghorban R, Alipour S, Tahmasebi S, Jokar A. The effect of vitamin D and E vaginal suppositories on tamoxifen-induced vaginal atrophy in women with breast cancer. *Support Care Cancer*. 2019;**27**: 1325-34.
[18] Lee YK, Chung HH, Kim JW, Park NH, Song YS, Kang SB. Vaginal pH-balanced gel for the control of atrophic vaginitis among breast cancer survivors: a randomized controlled trial. *Obstet Gynecol*. 2011;**117**: 922-27.
[19] Loprinzi CL, Abu-Ghazaleh S, Sloan JA, et al. Phase III randomized double-blind study to evaluate the efficacy of a polycarbophil-based vaginal moisturizer in women with breast cancer. *J Clin Oncol*. 1997;**15**: 969-73.
[20] Melisko ME, Goldman ME, Hwang J, et al. Vaginal Testosterone Cream vs Estradiol Vaginal Ring for Vaginal Dryness or Decreased Libido in Women Receiving Aromatase Inhibitors for Early-Stage Breast Cancer: A Randomized Clinical Trial. *JAMA Oncol*. 2017;**3**: 313-19.
[21] Mension E, Alonso I, Anglès-Acedo S, et al. Effect of Fractional Carbon Dioxide vs Sham Laser on Sexual Function in Survivors of Breast Cancer Receiving Aromatase Inhibitors for Genitourinary Syndrome of Menopause: The LIGHT Randomized Clinical Trial. *JAMA Netw Open*. 2023;**6**: e2255697.
[22] Pfeiler G, Glatz C, Königsberg R, et al. Vaginal estriol to overcome side-effects of aromatase inhibitors in breast cancer patients. *Climacteric*. 2011;**14**: 339-44.
[23] Quick AM, Zvinovski F, Hudson C, et al. Fractional CO2 laser therapy for genitourinary syndrome of menopause for breast cancer survivors. *Support Care Cancer*. 2020;**28**: 3669-77.
[24] Witherby S, Johnson J, Demers L, et al. Topical testosterone for breast cancer patients with vaginal atrophy related to aromatase inhibitors: a phase I/II study. *Oncologist*. 2011;**16**: 424-31.
